# Risk estimation for recurrent cardiovascular events using the SMART-REACH model and direct inpatient cost profiling in Indonesian ASCVD patients: a large-scale multicenter study

**DOI:** 10.3389/fcvm.2024.1425703

**Published:** 2024-08-02

**Authors:** Bambang Dwiputra, Dwita Rian Desandri, Anggoro Budi Hartopo, Dafsah Arifa Juzar, Amir Aziz Alkatiri, Naufal Zuhdi, Putra Andito Ramadhan, Bernadhet Daisy Kenconosari, Jason Phowira, Bambang Widyantoro

**Affiliations:** ^1^ Department of Cardiology and Vascular Medicine, Faculty of Medicine, Universitas Indonesia—National Cardiovascular Centre Harapan Kita, Jakarta, Indonesia; ^2^ Department of Cardiology and Vascular Medicine, Faculty of Medicine, Public Health, and Nursing, Universitas Gadjah Mada—Dr. Sardjito Hospital, Yogyakarta, Indonesia

**Keywords:** coronary heart disease, atherosclerotic cardiovascular disease, risk estimation, secondary prevention, cost profiling

## Abstract

**Introduction:**

With atherosclerotic cardiovascular disease (ASCVD) cases increasing in Indonesia, there is a growing need to identify high-risk patients for recurrent cardiovascular events. Risk stratification could guide optimal secondary preventive therapy. Understanding the ASCVD direct inpatient costs could further provide insight in reducing the economic burden that comes with Indonesia's high number ASCVD cases. However, there is a significant gap in Indonesian large-scale research on both of these valuable data. Employing the SMART-REACH model, we can profile the risk of recurrent cardiovascular events in Indonesian ASCVD patients.

**Objectives:**

Utilize the SMART-REACH model to estimate 10-year and lifetime risk of cardiovascular events in Indonesian ASCVD patients and describe the direct inpatient cost of ASCVD.

**Methods:**

This descriptive cross-sectional study gathered data from 3,209 ASCVD patients aged 45–80 from two major cardiovascular centers using purposive sampling. Participants were patients admitted between January 2020 and March 2023 with ST-elevated myocardial infarct (STEMI), non-ST-elevated myocardial infarct (NSTEMI), and chronic coronary syndrome (CCS) requiring elective percutaneous coronary intervention (PCI). The SMART-REACH risk estimation model required clinical data upon admission, laboratory results within the first 24 h of admission, and cardiovascular medication prescribed upon discharge. The SMART-REACH model is a Fine and Gray competing risk model incorporating cardiovascular risk factors that estimates individual 10-year and lifetime risk for recurrent cardiovascular events which includes myocardial infarction, stroke, or vascular death. Direct inpatient cost profiling totaled all medical expenses incurred from ASCVD diagnosis admission to discharge. Results were reported descriptively with subgroup analyses.

**Results:**

The cohorts (mean age 60.15 ± 8.6 years) were predominantly male [*n* = 2,537 (79.1%)], hypertensive [*n* = 2,267 (70.6%)], and diagnosed with STEMI [*n* = 1,732 (54%)]. The SMART-REACH model calculated a mean 10-year risk of 30.2% (95% CI 29.7–30.6) and a lifetime risk of 62.5% (95% CI 62.1–62.9). The direct inpatient cost of ASCVD patients includes a median 3,033 USD, with highest median costs in the STEMI subgroup (3,270 USD).

**Conclusions:**

A significant number of Indonesian ASCVD patients exhibited notably high 10-year and lifetime risks of experiencing a major cardiovascular event. Combined with the direct inpatient cost, therapy optimization is crucially needed to mitigate these risks and further cost burden.

## 1 Introduction

Cardiovascular diseases (CVD) are the leading cause of death globally and have become a major health problem across the world. The World Health Organization (WHO) has reported more than 17 million deaths globally due to CVD in 2015 (1). National Basic Health Research in Indonesia also reported that the prevalence of CVD in Indonesia has been increasing from 0.5% in 2013 to 1.5% in 2018 (2). This number might continue to rise as cases of diabetes mellitus (DM) and hypertension, both risk factors for cardiovascular disease coupled with cholesterol-related risk factors, have also increased. Cases of DM increased from 14.8% in 2013 to 21.8% in 2018, and hypertension increased from 26% in 2013 to 34% in 2018 (3).

Atherosclerotic cardiovascular disease (ASCVD) has posed an increasing burden on the healthcare system for decades. It is projected that the cost of ASCVD will increase by over 2.5-fold from 2015 to 2035 (4). This cost burden has significant consequences not only for payers but also for patients and healthcare providers. Identifying patients at high risk of ASCVD is becoming increasingly important; risk stratification could help clinicians determine which patients benefit most from innovative and often costly therapy (5). For patients, information regarding their risk is crucial for prognosis and future decisions regarding preventive treatment. One risk stratification method is the SMART-REACH model, developed in 2018, which can estimate the 10-year risk and lifetime risk for myocardial infarction, stroke, or vascular death in individual patients with clinically manifest ASCVD (6).

This study aims to assess the risk of recurrent cardiovascular events among Indonesian ASCVD patients in terms of 10-year and lifetime risk using the SMART-REACH model and to describe the direct inpatient cost burden of these patients. The results will be used as evidence to strengthen population health programs to prevent and control secondary ASCVD.

## 2 Materials and methods

### 2.1 Study design and population

This is a descriptive, non-interventional study conducted using retrospective and cross-sectional data extracted from medical records at two tertiary referral hospitals in Indonesia, which are National Cardiovascular Center Harapan Kita (NCCHK), Jakarta and Dr. Sardjito Hospital, Yogyakarta. The subjects of this study comprised patients admitted with ASCVD at these hospitals between January 2020 up to March 2023. Inclusion and exclusion criteria were applied to purposively include eligible patients in the dataset. Inclusion criteria encompassed patients aged 45–80 years admitted with a diagnosis of ST-elevated myocardial infarct (STEMI) (ICD-10: I21), non- ST-elevated myocardial infarct (NSTEMI) (ICD-10: I21), or chronic coronary syndrome (CCS) requiring elective PCI (ICD-10: I25.1) who were alive at discharge. Exclusion criteria comprised patients lacking adequate registry data necessary for risk calculation, as well as patients with terminal malignancy, end-stage renal disease, and hepatic cirrhosis. These criteria were based on those of the REACH and SMART cohorts (7, 8). Ethical clearance for the study was obtained from the local institutional review boards in both hospitals.

### 2.2 Data collection

**Figure 1:**
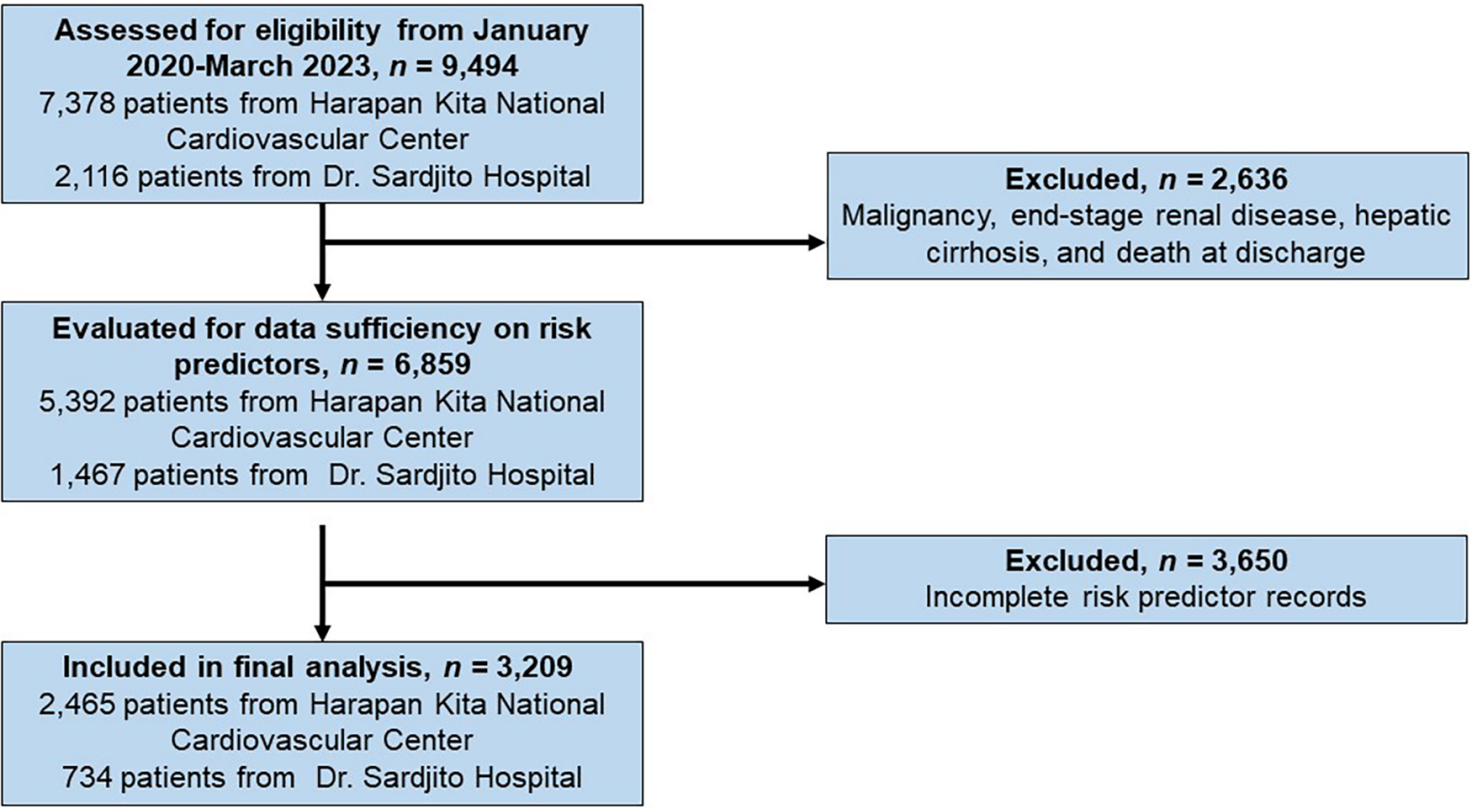
The flow diagram for patient selection and eligibility.

Data collection involved purposive sampling from secondary sources, specifically electronic medical and billing records from both hospitals. Subjects were identified from both the One ACS Registry and the Indonesia PCI Registry (9, 10). Upon applying the inclusion and exclusion criteria, 3,209 eligible patients were selected from an initial pool of 9,494 patients for the present analysis (Figure 1). To ascertain the risk score for each patient, we examined clinical information collected upon admission, including age, gender, smoking habits, presence of diabetes mellitus (DM), previous history of vascular disease, heart failure, or atrial fibrillation. Additionally, lipid values obtained within the initial 24 h of hospitalization, baseline serum creatinine levels, baseline HbA1c levels, and medications prescribed at discharge once the patient was deemed clinically stable were taken into consideration. The medications considered in the calculation included statins, ezetimibe, PCSK9 inhibitors, antithrombotic treatments, GLP1 receptor agonists, SGLT2 inhibitors, and low-dose colchicine. The data pertaining to direct inpatient costs only encompassed the total direct medical expenses incurred during hospitalization for ASCVD diagnosis, from patient admission to discharge.

### 2.3 The SMART-REACH model

The SMART-REACH risk model is a competing-risk adjusted Fine and Gray model, designed for estimating both 10-year and lifetime risk of major cardiovascular events and non-cardiovascular mortality in patients with clinically manifest vascular disease. The underlying model formulas and methodology were detailed in the original SMART-REACH publication. Using age as the underlying timescale, life tables are constructed to calculate risks for every 1-year interval, starting from the individual's initial age and extending up to a maximum age of 90 years. The model was derived using adapted Fine and Gray models to accommodate left truncation and right censoring. It incorporates several predictors, including age, sex, current smoking status, diabetes mellitus (DM), history of heart failure, history of atrial fibrillation, systolic blood pressure (BP), serum creatinine concentration, number of locations of cardiovascular disease (cerebrovascular, coronary, and peripheral artery disease), as well as total and low-density lipoprotein cholesterol (LDL-C) levels (6).

### 2.4 Outcome

The outcome of this study is the 10-year and lifetime risk of recurrent cardiovascular events and associated direct inpatient cost in patients diagnosed with ASCVD. The cardiovascular events considered include myocardial infarction, stroke, and cardiovascular death. Descriptive reporting is used to present direct inpatient costs, focusing on total direct medical expenses. Additionally, further analyses of 10-year and lifetime risk are conducted within different patient subgroups. These subgroups account for various risk factors such as hypertension, diabetes mellitus (DM), and active smoking, as well as differences in lipid-lowering treatments and antiplatelet therapy usage in relation to the risk profile of recurrent cardiovascular events. Regarding direct inpatient costs, subgroup analyses examine differences between genders, diagnoses, and cardiovascular centers.

### 2.5 Statistical analysis

A descriptive statistical approach is utilized to outline the baseline characteristics and estimated risk within the study subgroups. Prior to analysis, the collected data underwent filtering, sorting, and cleaning procedures to ensure uniformity and data quality. Baseline characteristics of eligible patients, including age, sex, smoking status, diagnosis, past medical history, physical examination and laboratory measurements, and medication usage, are included. Frequency distributions and mean with standard deviation are used to illustrate these characteristics. Additionally, the estimated risk of recurrent cardiovascular events within the study subgroups is calculated as the mean with a 95% confidence interval (CI) derived from the SMART-REACH calculator. Direct inpatient cost data are presented in terms of overall cost, with mean, median, standard deviation, 95% CI, and percentiles provided for clarity. All statistical analysis used SPSS version 22 (IBM, USA).

## 3 Results

### 3.1 Patients baseline characteristics

Out of the total of 3,209 patients, 30.5% of patients were active smokers upon admission. Among the diagnoses, the majority of cases were admitted with STEMI [*n* = 1,732 (54.0%)], followed by NSTEMI [*n* = 904 (28.2%)] and CCS elected for PCI [*n* = 573 (17.9%)], respectively. The subjects' medical histories encompassed various conditions including hypertension (70.6%), DM (36.7%), hypercholesterolemia (30.7%), cerebrovascular disease (4.6%), peripheral artery disease (0.9%), heart failure (13.8%), and atrial fibrillation (3.1%). HbA1c data was available for 2,345 patients, representing 73% of the total subjects in the study (Table 1). This limited data collection occurred because, according to both hospitals' standard operating procedures, HbA1c tests were only performed on patients with a known history of type 2 DM or elevated fasting blood sugar upon admission. However, the SMART-REACH model calculation only requires HbA1c for assessing the risk of diabetic patients, whereas non-diabetic patients do not require HbA1c data for the calculation of their cardiovascular risk. Consequently, complete HbA1c data is available for all diabetic patients, while all the absent HbA1c data in this study pertains to the subgroup without DM.

**Table 1:** Baseline characteristic of the study.

Total, *n* (%)	3,209 (100)
Male, *n* (%)	2,537 (79.1)
Age (years)^a^	60.15 ± 8.6
Active smoker, *n* (%)	979 (30.5)
Coronary artery disease diagnosis	
STEMI, *n* (%)	1,732 (54.0)
NSTEMI, *n* (%)	904 (28.2)
CCS, *n* (%)	573 (17.9)
Past medical history	
Hypertension, *n* (%)	2,267 (70.6)
Diabetes mellitus, *n* (%)	1,178 (36.7)
Hypercholesterolemia, *n* (%)	984 (30.7)
Cerebrovascular disease, *n* (%)	148 (4.6)
Peripheral artery disease, *n* (%)	28 (0.9)
Heart failure, *n* (%)	444 (13.8)
Atrial fibrillation, *n* (%)	100 (3.1)
Previous revascularization	
PCI	504 (15.7)
CABG	74 (2.3)
Family history of premature CVD	286 (8.9)
Physical examination and laboratory measurements	
Weight^a^	67.1 ± 12.0
BMI^a^	25.0 ± 3.7
Systolic blood pressure (mmHg)^a^	133.4 ± 26.6
Diastolic blood pressure (mmHg)^a^	77.99 ± 15.8
Heart rate^a^	81.5 ± 21.2
Total cholesterol (mg/dl)^a^	172.6 ± 43.816
LDL-C (mg/dl)^a^	115.2 ± 39.223
Creatinine (mg/dl)^a^	1.2 ± 0.5
HbA1c (mg/dl)^a^ *(n = 2,345)*^b^	7.03 ± 1.96
Within DM subgroup *(n = 1,178)*	8.2 ± 2.08
Without DM subgroup *(n = 1,168)*	5.81 ± 0.62
EF^a^	45.6 ± 12.1
Medication at discharge	
Lipid lowering medication, *n* (%)	3,018 (94)
Simvastatin, *n* (%)	953 (29.8)
Atorvastatin, *n* (%)	2,024 (63.1)
Rosuvastatin, *n* (%)	41 (1.3)
Antiplatelet therapy, *n* (%)	2,900 (90.4)
Aspirin only, *n* (%)	111 (3.5)
Dual antiplatelet therapy, *n* (%)	2,789 (86.9)
Colchicine, *n* (%)	18 (0.6)

BMI, body mass index; CABG, coronary artery bypass grafting; CCS, chronic coronary syndrome; CVD, cardiovascular disease; DM, diabetes mellitus; EF, ejection fraction; LDL-C, low density lipoprotein cholesterol; NSTEMI, non-ST-elevation myocardial infarction; PCI, percutaneous coronary intervention; STEMI, ST-elevation myocardial infarction.

^a^ Data are displayed as mean (standard deviation).

^b^ HbA1c measurement was restricted to patients with a documented history of type 2 diabetes mellitus (T2DM) or hyperglycemia on admission.

### 3.2 Risks of recurrent cardiovascular events

**Figure 2:**
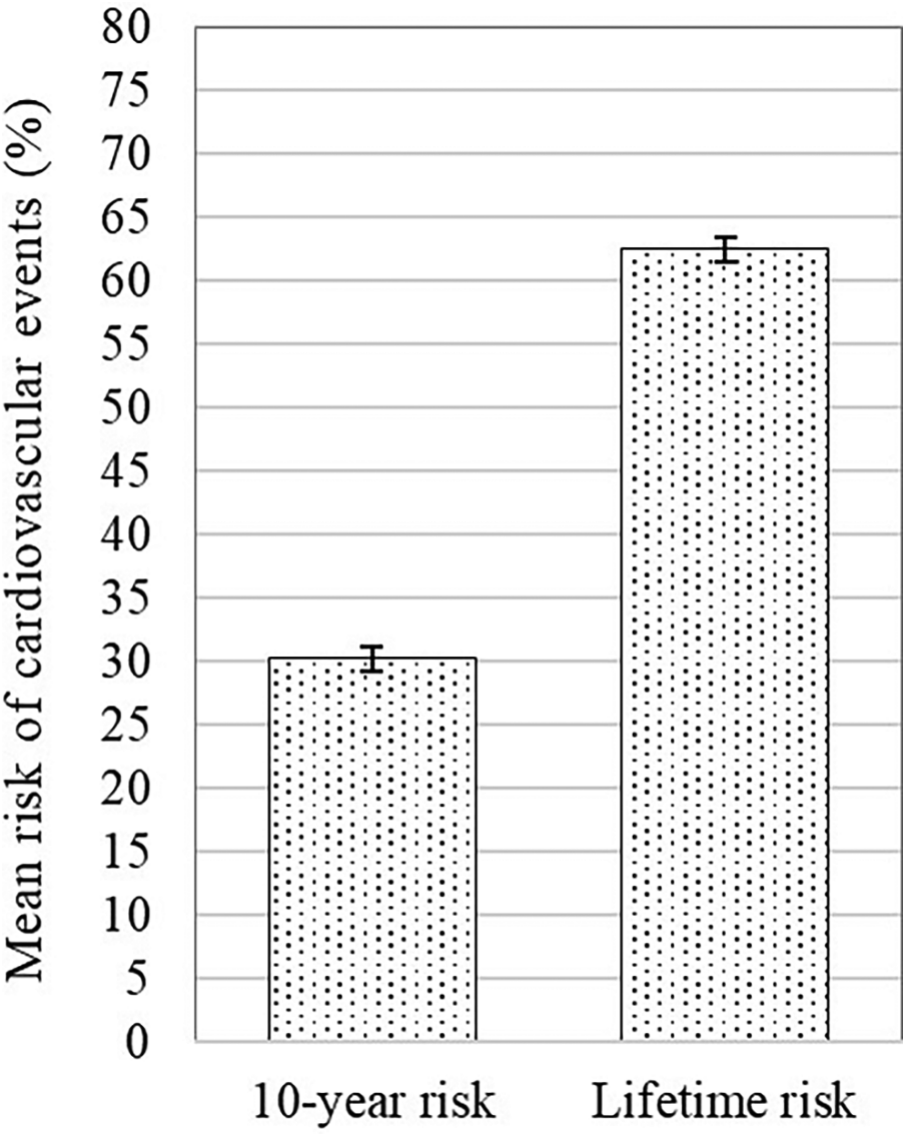
Mean 10-year risk of cardiovascular events with 95% confidence interval of the total sample.

**Figure 3:**
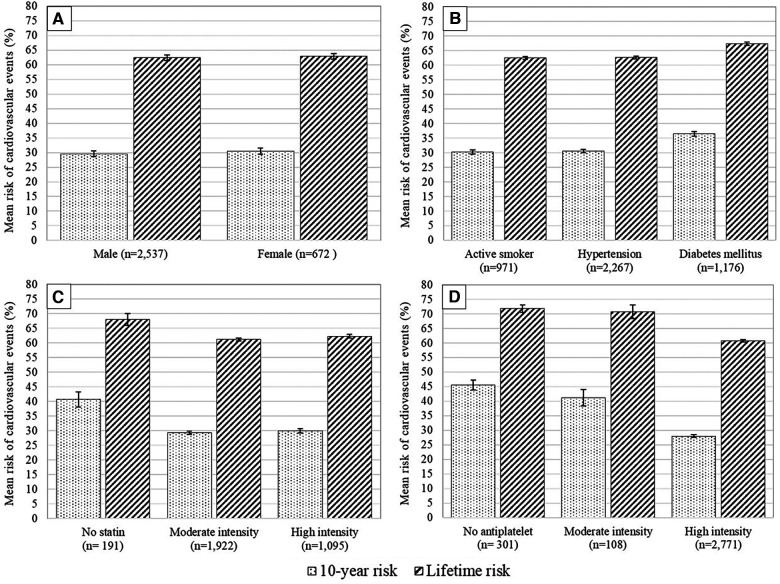
Mean 10-year risk of cardiovascular events with 95% confidence interval based on different patients’ subgroups: (**A**) sex, (**B**) risk factors, (**C**) statin intensity given at discharge, and (**D**) antiplatelet therapy given at discharge.

This study found an average 10-year risk of recurrent cardiovascular events of 30.2% (95% CI 29.7–30.6%), and a lifetime risk averaging 62.5% (95% CI 62.1–62.9%) (Figure 2). Subgroup analysis demonstrates differences in ASCVD risk profiles. Female patients exhibited a higher average risk than males (10-year: mean 30.5% vs. 29.6%; lifetime: mean 62.9% vs. 62.4%). Patients with diabetes mellitus (DM) had the highest average risk for both 10-year and lifetime events compared to active smokers and hypertensive subgroups (10-year: mean 36.5% vs. 30.3% vs. 30.4%; lifetime: mean 67.4% vs. 62.5% vs. 61.7%) (Figures 3A,B).

Upon discharge, nearly equal proportions of patients received simvastatin 20 mg (29.5%), atorvastatin 20 mg (30%), or atorvastatin 40 mg (32%), with a small portion (6%) not receiving a statin. Most patients receiving statins (59.9%) were prescribed moderate-intensity statins (e.g., atorvastatin 20 mg) rather than high-intensity statins (36.5%). Patients on atorvastatin 20 mg had the lowest average risk of recurrent cardiovascular events compared to those on atorvastatin 40 mg and simvastatin 20 mg, for both 10-year (28.4% vs. 29.8% vs. 30.1%) and lifetime risks (61.2% vs. 62.2% vs. 62.1%). Additionally, patients treated with moderate-intensity statins showed lower average risks compared to those on high-intensity statins (10-year: mean 29.3% vs. 29.9%; lifetime: 61.2% vs. 62.3%) (Figure 3C).

The majority of patients (86.4%) received dual antiplatelet therapy (DAPT), with others receiving aspirin only (3.4%) or no antiplatelet therapy (9.4%). Patients on DAPT had a significantly lower risk than those on aspirin only or no antiplatelet therapy (10-year: 28.0% vs. 41.2% vs. 45.6%; lifetime: 60.7% vs. 70.7% vs. 71.8%) (Figure 3D).

### 3.3 Direct inpatient cost of ASCVD

The median direct inpatient cost of all 3,209 patients was 3,033 USD (IQR 1,573 USD). Male and female patients showed relatively similar median expenses [3,043 USD (IQR 1,527 USD) vs. 2,956 USD (IQR 1,727 USD)]. Among the diagnosis groups, STEMI patients had the highest median direct inpatient cost compared to the NSTEMI and CCS patients (3,643 USD vs. 3,768 USD vs. 2,828 USD). Since all CCS patients in this study were elected for PCI, their length of stay and procedures were similar, resulting in a lower interquartile range (IQR 760 USD). In contrast, NSTEMI patients showed the most varied direct inpatient cost between patients (IQR 2,922 USD). Lastly, patients admitted to the National Cardiovascular Center Harapan Kita had a higher median direct inpatient cost compared to those admitted to Dr. Sardjito Hospital, [3,769 USD (IQR 1,716 USD) vs. 2,712 USD (IQR 888 USD)] (Table 2).

**Table 2:** Total indirect inpatient cost of study subjects.

	Overall population (*n* = 3,209)	Sex		ASCVD diagnosis			CV center	
		Male (*n* = 2,537)	Female (*n* = 672)	STEMI (*n* = 1,732)	NSTEMI (*n* = 904)	CCS (*n* = 573)	NCCHK (*n* = 2,475)	Dr. Sardjito Hospital (*n* = 734)
Median	3,033	3,043	2,956	3,270	2,491	2,696	3,135	2,689
Standard deviation	3,164	3,006	3,700	2,612	4,506	1,596	3,450	1,597
25th percentile	2,285	2,333	2,100	2,698	1,140	2,307	2,281	2,301
75th percentile	3,858	3,861	3,827	4,003	4,062	3,067	3,998	3,189
Interquartile range	1,573	1,527	1,727	1,304	2,922	760	1,716	888

ASCVD, atherosclerotic cardiovascular disease; CCS, chronic coronary syndrome; CI, confidence interval; CV, cardiovascular; NCCHK, National Cardiovascular center Harapan Kita; NSTEMI, non-ST-elevation myocardial infarction; STEMI, ST-elevation myocardial infarction. Currency in USD with conversion rate of 1 USD = 15.734 IDR.

## 4 Discussion

In this study, we profiled the 10-year and lifetime recurrent ASCVD risk from two major cardiovascular centers in Indonesia. Our study highlighted the remarkably elevated 10-year and lifetime risk of experiencing major cardiovascular events among the population of Indonesian ASCVD patients. Approximately 3 out of 10 individuals were projected to experience recurrent ASCVD within the span of 10 years, with about twice as many expected to encounter such an event over their lifetime. These risks were particularly higher among female subjects, and also among diabetic individuals compared to smoking or hypertensive patients. STEMI patients demonstrated the highest direct inpatient cost, followed by NSTEMI and CCS patients, suggesting that the acute nature of acute coronary syndrome (ACS) may result in greater associated costs during hospitalization. Given the substantial expenses associated with hospitalization in CVD patients, utilizing this model could be beneficial to help optimize therapy in patients at higher risk and to provide an estimate of future ASCVD burden.

To our knowledge, this is the first study in Asia to utilize the SMART-REACH model for estimating recurrent ASCVD risk among the secondary-prevention population. A study by van Trier et al., conducted on the European population, indicated a ten-year and lifetime risk of 26% and 55% respectively (*n* = 416, mean (SD) age 65 (9) years, and 80% men) (11). Similarly, Siniawski et al. observed a ten-year risk of 34.95% in the Argentinian population (*n* = 296, mean (SD) age 68.2 (9.4) years, and 75.7% men) (12). Discrepancies in CVD recurrent risk estimation between these studies and ours could be attributed to differences in the incidence rate of CVD and its risk factors such as hypertension, dyslipidemia, and diabetes among CVD patients.

In Indonesia, several other studies have reported their findings on ASCVD risk assessments. A study estimated CVD risk using the WHO/ISH risk charts and reported that about two-thirds of the population exhibited a CVD risk below 10%. However, the study mainly focused on primary prevention risk assessment and recruited only 3.4% of participants with a previous history of CVD (13). Another study utilizing the SMART2 algorithm to estimate the risk of recurrent coronary heart disease (CHD) demonstrated that 65% of the enrolled patients possessed a very high risk of 10-year recurrent CHD equal to or exceeding 30%. It is noteworthy that this latter study involved a more limited cohort of 395 participants (14). Moreover, the study population was localized to the eastern region of Indonesia, thus the findings may not be fully representative of the entire country.

Approximately 70% of the participants in this investigation presented with hypertension, while 36% were diagnosed with diabetes mellitus, highlighting the importance of these conditions as significant risk factors within this population. Thus, one of the important laboratory parameters assessed was HbA1c. The study observed that the average HbA1c values within the subgroup of diabetes mellitus were 8.2%, surpassing the recommended target range. This could be attributed to the inclusion of newly diagnosed diabetic patients within the subgroup. Moreover, given this study's cross-sectional nature, it is important to note that this finding cannot demonstrate a therapeutic response. A study conducted by Zhang et al., has revealed that individuals with Type 2 Diabetes Mellitus (T2DM) and moderate baseline ASCVD risk, face a significantly increased cardiovascular risk if their HbA1c levels range between 7.0% to 8.0% (15).

Our subsequent subgroup analysis revealed a varied distribution of recurrent ASCVD risks across various patient subgroups. We found higher 10-year and lifetime risks among individuals with diabetes mellitus compared to the actively smoking and hypertensive patients. An observational study among type 1 diabetes mellitus patients with a median follow-up of 29 years reported HbA1c as the strongest modifiable risk factor for the first and subsequent CVD events. Each 1% increase in mean HbA1c is associated with a 28% increased risk of developing subsequent CVD events and an 89% increased risk of encountering recurrent major adverse cardiovascular events (16). Therefore, a controlled glycemic level can significantly lower the risk of recurrent events. This information can guide more intensified treatment options for those with a higher risk of ASCVD recurrence. Given that ischemic heart disease is associated with high unit costs and financial burden, identifying and managing patients at higher risks (diabetic, hypertensive, actively smoking), especially in populations with limited access to high-quality healthcare, remains essential in lowering the CVD burden in these vulnerable populations. This also highlights the significance of devising tailored, intensive preventive strategies in ASCVD secondary prevention.

In the present study, not all patients received guideline-directed medical therapy for secondary prevention. Despite the American Heart Association/American College of Cardiology/multisociety (AHA/ACC/MS) and European Society of Cardiology (ESC) guidelines recommending the use of high-intensity statin for every patient with a history of ASCVD, only 34.1% of the included patients were prescribed high-intensity statin at discharge. This figure of high-intensity statin underutilization for very high-risk secondary prevention patients is comparable with other studies in different countries (12, 17–19). In this study, individuals receiving statins of higher intensity showed elevated ASCVD risks compared to those receiving statins of moderate intensity, albeit to a marginal extent. Similar results were found between patients receiving 20 vs. 40 mg of atorvastatin. These findings could be attributed to a higher prevalence of risk factors among patients prescribed with the higher statin, consequently elevating the risk of recurrent ASCVD. This was confirmed by our further analysis revealing that among patients receiving high-intensity statin, not only did they demonstrate a significantly larger percentage of active smokers, but also higher total cholesterol, LDL-C, and HbA1c levels.

Some patients were not prescribed anti-thrombotic therapy and a bigger portion of the patients did not receive high-intensity statin. The use of additional drug strategies (i.e., colchicine, glucagon-like peptide-1 agonists/GLP-1a, and sodium–glucose cotransporter 2 inhibitors/SGLT2i) was not regularly prescribed in both hospitals where the study population was collected. Several studies have described the potential risk reduction of optimal guideline-directed preventive ASCVD therapy using the SMART-REACH model, which yielded a median risk reduction of 6%–10% and 9%–20% for 10-year and lifetime risk respectively (11, 20). In Indonesia, according to the Indonesian Case Base Groups (INA-CBGs), high-intensity statin is only covered by the government during hospitalization until 3 weeks post-discharge. A cost-effectiveness analysis revealed that the use of high-intensity statin post-hospitalization in ACS patients had an incremental cost-effectiveness ratio of 31.843.492.98 IDR (USD 2,024) per quality-adjusted life year compared to conventional-dose statin. This finding advocates the prescription of high-intensity over low to moderate-intensity statin as a cost-effective secondary prevention strategy to mitigate recurrent ASCVD over a prolonged time period (21).

It is of interest that our finding revealed the highest direct inpatient cost in STEMI subjects compared to patients with NSTEMI or CCS. An observational study enrolling 218 hospitals in Asia reported several significant predictors of high-cost care for ACS, including age, male sex, prior disease history, previous history of hospitalization in the last 3 months, having an invasive procedure, hospital type, and longer hospital stay (22). The higher cost observed among STEMI patients in our study could be attributed to a combination of those factors, however, this finding further confirms the high economic burden posed by CVD. When compared to other Asian countries, China was reported to incur the highest cost for hospitalized ACS patients (STEMI mean cost: USD 7,790; NSTEMI: USD 7,450), more than twice the direct inpatient cost that we presented in our study. Thailand also presented a higher cost of hospitalization averaging at USD 4,427 for STEMI and USD 3,321 for NSTEMI. The direct inpatient cost in our study was comparable to figures documented in Hong Kong and slightly surpassed those reported in Vietnam. Nonetheless, it is crucial to emphasize the distinction that in Hong Kong, the government subsidizes all emergency admissions for ACS, leading to generally low healthcare payments for patients (22).

Moreover, with an average cost of approximately Rp55 million (approximately 3,500 USD) per hospital discharge, it may be important to note that our study specifically focused on two tertiary referral hospitals in Indonesia, resulting in relatively higher costs. However, the choice of tertiary referral hospitals is justified by the superior quality of care they offer, potentially making the Rp55 million figure indicative of the optimal cost of disease care. Considering a 10-year risk of recurrent ACS at 30.2%, the significance of timely secondary preventive treatment becomes evident, especially since ischemic heart disease represents the highest expenditure (9.65% in 2016) within the Indonesian National Health Insurance (JKN) (23). In the context of secondary prevention, one study observed that even among patients categorized as having a high risk for CVD, healthcare costs were three times higher in those with a history of cardiovascular events like ACS (24). Furthermore, it is important to note that this analysis does not encompass the indirect costs of healthcare, which have been identified as significant contributors to further economic burden and productivity loss in various studies.

## 5 Limitations

The limitations of the ASCVD risk calculation using the SMART-REACH model have been previously discussed (6). Most existing risk prediction tools lack sufficient calibration for the Asian population, including the SMART-REACH model, which has yet to be validated for any population group other than Western European and North American. Furthermore, the SMART risk score, which the SMART-REACH model is derived from, has been updated to the SMART2 risk score in order to match the external validations done after its conception (25). This study exclusively included patients with STEMI, NSTEMI, and CCS, while omitting other coronary heart diseases, cerebrovascular disease, peripheral artery disease, and alternative forms of vascular disease. The inclusion of diverse atherosclerotic manifestations would have provided additional insights into the prevalence of recurrent CVD in Indonesia. Many diabetic ASCVD patients in this study have also been excluded since HbA1c is not regularly checked at both hospitals during admission, rendering the data unable to be calculated using the SMART-REACH model. Furthermore, due to administrative challenges in data collection, only total costs were attainable from both cardiovascular centers, excluding the breakdown of individual cost components, such as medication and procedure bills. This is a missed opportunity, as such details could have provided valuable insights, such as healthcare cost differences between patients receiving different antiplatelet medications at discharge.

## 6 Conclusions

By employing the SMART-REACH model, a significant number of Indonesian ASCVD patients was anticipated to exhibit considerably elevated risks of experiencing a major cardiovascular event over both a 10-year and lifetime period. Notably, patients with STEMI incurred the highest direct inpatient costs, followed by those with NSTEMI and CCS. These findings underscore the imperative of identifying those at a heightened risk of recurrent ASCVD, as optimizing therapy and implementing tailored secondary prevention measures could lead to substantial health and financial benefit.

## Data Availability

The raw data supporting the conclusions of this article will be made available by the authors, without undue reservation.
